# ALKBH5‐Driven m6A Demethylation Boosts Inflammation and Autophagy in LPS‐Stimulated Macrophages

**DOI:** 10.1002/iid3.70348

**Published:** 2026-03-16

**Authors:** Gui Wang, Ting Zhou, Shujun Zhou

**Affiliations:** ^1^ Department of Critical Care Medicine, The First People's Hospital of Changzhou The Third Affiliated Hospital of Soochow University Changzhou China

**Keywords:** Acute respiratory distress syndrome, AlkB homolog 5, alveolar macrophage, inflammatory response, N6‐methyladenosine

## Abstract

**Purpose:**

Acute Respiratory Distress Syndrome (ARDS) remains a critical health threat with limited pharmacological treatments. This study investigates the role of the N^6^‐methyladenosine (m^6^A) demethylase AlkB homolog 5 (ALKBH5) in alveolar macrophages and its subsequent impact on inflammation and autophagy during ARDS pathogenesis.

**Methods:**

Primary mouse alveolar macrophages were stimulated with Lipopolysaccharide (LPS) and transfected with ALKBH5 knockdown or overexpression plasmids. Global m6A levels were assessed via Dot Blot, while m^6^A modification of the target gene ULK1 was analyzed using MeRIP‐qPCR. Macrophage polarization, migration, and autophagy (LC3‐II/p62 flux) were evaluated *in vitro*. The therapeutic potential of ALKBH5 downregulation was validated in an LPS‐induced murine ARDS model through lung histology, cytokine analysis (ELISA), and microvascular permeability assessments.

**Results:**

ALKBH5 was found to be a critical regulator of m^6^A methylation in alveolar macrophages. LPS stimulation decreased m^6^A modification of ULK1 mRNA, a process reversed by ALKBH5 downregulation. ALKBH5 knockdown significantly suppressed LPS‐induced autophagy by reducing autophagosome formation and inhibited M1 pro‐inflammatory polarization. In vivo, ALKBH5 downregulation significantly mitigated lung tissue damage, reduced pulmonary edema, and lowered levels of pro‐inflammatory cytokines, including IL‐1β, TNF‐α, and IL‐17.

**Conclusions:**

Our findings demonstrate that ALKBH5‐driven m^6^A demethylation of ULK1 exacerbates ARDS by promoting macrophage autophagy and pro‐inflammatory responses. These results suggest that targeting ALKBH5 may disrupt pathogenic m6A demethylation, offering a novel therapeutic strategy for mitigating ARDS progression.

## Graphical abstract

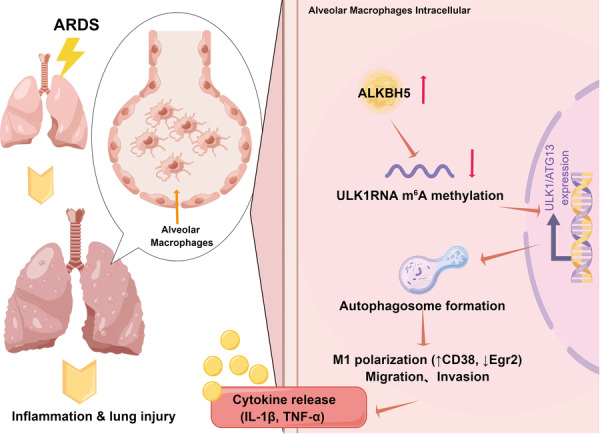

## 1 Introduction

Acute Respiratory Distress Syndrome (ARDS) is a life‐threatening critical illness characterized by non‐cardiogenic pulmonary edema and refractory hypoxemia, triggered by diverse pulmonary (e.g., pneumonia, aspiration) or extrapulmonary (e.g., sepsis, trauma) insults [1, 2, 3]. Recent global epidemiological studies indicate that ARDS accounts for 10.4% of ICU admissions, with mortality risk strongly correlated to disease severity: 34.9% for mild, 40.3% for moderate, and 46.1% for severe ARDS [4, 5]. Alarmingly, an estimated 40% of ARDS cases remain undiagnosed due to limited clinical recognition, suggesting true incidence may be substantially higher [6, 7, 8]. The COVID‐19 pandemic has exacerbated this burden, with severe cases frequently progressing to ARDS and mortality rates approaching 50% in mechanically ventilated patients [9]. In specific populations such as severe traumatic brain injury (sTBI) patients, ARDS development significantly worsens outcomes, with mortality exceeding 37% in worsening cases. Despite advances in supportive care (e.g., lung‐protective ventilation, prone positioning) [10], ARDS continues to pose a major global health threat, underscoring the urgent need for deeper mechanistic insights and targeted therapies.

In the pathogenesis of ARDS, infections caused by pathogenic microorganisms are the most common etiological factors [11, 12, 13, 14, 15, 16]. Immune cell reactions triggered by these infections or injuries lead to the release of numerous inflammatory mediators, resulting in an uncontrollable cascade of inflammatory responses that damage alveolar epithelial and capillary endothelial cells [17]. Currently, treatment strategies for ARDS primarily involve lung‐protective ventilation, fluid management, immune enhancement, and organ support, but there is a notable lack of specific pharmacological therapies [18, 19, 20, 21, 22]. Given the pressing need for effective ARDS treatments, it is essential to conduct in‐depth research into the molecular mechanisms that amplify the inflammatory response in infection‐induced ARDS [23, 24, 25, 26]. Identifying novel targets to suppress uncontrolled inflammatory responses could pave the way for precision therapies for ARDS.

N6‐methyladenosine (m6A) is the most prevalent internal modification of messenger RNA (mRNA) and plays a crucial role in regulating various mRNA functions [27]. These m6A modifications are commonly found in precursor mRNAs, particularly around stop codons and 3' untranslated regions (UTRs) [28]. A recent analysis of the m6A transcriptome has revealed that m6A predominantly occurs within the RRACU consensus motif in mammals. The addition and removal of m6A modifications are mediated by a methyltransferase complex (commonly referred to as the “writer”) and demethylases (known as “erasers”), respectively [29, 30, 31, 32]. The methyltransferase complex consists of methyltransferase‐like 3 and 14 (METTL3 and METTL14), along with cofactors such as Wilms tumor 1‐associated protein (WTAP), VIRMA (KIAA1429), and RNA‐binding motif protein 15 (RBM15) [33]. Additionally, fat mass and obesity‐related protein (FTO) and AlkB homolog 5 (ALKBH5) function as demethylases, oxidatively removing m6A with the assistance of α‐ketoglutarate as a substrate and Fe (II) as a coenzyme [34, 35]. FTO is also involved in the demethylation of N6,2'‐O‐dimethyladenosine (m6Am), while ALKBH5 specifically targets m6A for demethylation [36]. Moreover, reader proteins that recognize m6A play critical roles in various biological processes [37, 38]. For example, YT521‐B homology (YTH) domain‐containing proteins, YTHDC1 and YTHDC2, promote alternative splicing and facilitate mRNA export from the nucleus to the cytoplasm. Heterogeneous nuclear ribonucleoprotein G (HNRNPG) modulates RNA structures through interactions with RNA [39, 40]. YTH domain family 1 (YTHDF1), YTHDF3, METTL3, and eukaryotic initiation factor 3 (eIF3) regulate translation efficiency. Additionally, insulin‐like growth factor 2 mRNA‐binding proteins (IGF2BPs), YTHDF2, YTHDF3, and YTHDC2 influence mRNA stability [41, 42, 43]. Recently, there has been growing interest in the role of epigenetic modifications, particularly N6‐methyladenosine (m6A) methylation, in regulating the inflammatory response associated with ARDS [44]. m6A methylation, the most prevalent modification of mRNA, has been implicated in modulating various biological processes, including immune response and inflammation [45, 46, 47]. Understanding the interplay between m6A methylation and the inflammatory cascade in ARDS holds great promise for identifying novel therapeutic targets and developing precision medicine approaches for the management of this devastating syndrome [48, 49]. Emerging evidence suggests a potential association between m6A methylation and the development of macrophage function [39]. ALKBH5 was prioritized due to its established role in macrophage‐mediated inflammation [50] and its underexplored function in ARDS pathogenesis [51, 52, 53], whereas other demethylases like FTO have been more extensively studied in metabolic contexts. While ALKBH5 has been implicated in cancer and sepsis [54], its specific role in modulating alveolar macrophage autophagy and M1 polarization via m6A‐dependent regulation of ULK1 in ARDS has not been previously demonstrated, highlighting the unique contribution of this work.

Autophagy, a critical intracellular degradation process, plays a complex yet pivotal role in the pathogenesis of ARDS by modulating inflammatory responses and cell survival in alveolar macrophages. The initiation of autophagy is primarily governed by the ULK1 (Unc‐51 like autophagy activating kinase 1) protein kinase complex, wherein ULK1 interacts with its regulatory subunit ATG13 to transduce upstream signals to the downstream autophagy machinery. This ULK1–ATG13 axis serves as the crucial starting point for autophagosome formation. ULK1/ATG13‐mediated autophagy not only promotes cell survival but also influences macrophage polarization, potentially amplifying M1 pro‐inflammatory responses—a link we directly assess via LC3 flux, p62 degradation, and CD38/Egr2 staining. Given that dysregulated autophagy is increasingly recognized as a key contributor to the excessive inflammation and tissue damage observed in ARDS, we reasoned that key initiators of this pathway would be relevant downstream effectors of inflammatory signals. Therefore, we selected ULK1 and ATG13 as candidate targets to investigate whether their expression is modulated by ALKBH5‐mediated m6A demethylation, thereby potentially linking epitranscriptomic regulation to the core autophagy pathway in LPS‐induced macrophage inflammation.

In this study, we hypothesized that ALKBH5‐driven m6A demethylation exacerbates ARDS by promoting macrophage autophagy and inflammation. We aimed to: (1) Define ALKBH5's role in m6A/ULK1 regulation using MeRIP‐qPCR and Western blot; (2) Characterize its impact on autophagy (LC3/p62, GFP‐LC3 puncta) and polarization (flow cytometry); (3) Validate therapeutic potential in an LPS‐induced murine ARDS model via histology and ELISA.

## 2 Materials and Methods

### 2.1 Isolation and Culture of Mouse Alveolar Macrophage Cells

Macrophage isolation was performed with previous reported method [55]. Prepare a 1:200 dilution of CD45 antibody (#HY‐P80063, MCE, Shanghai, China) in PBS solution. Inject the antibody dilution into the tail vein of C57BL/6 mice (SLAC Animal Center, Shanghai, China) and let it circulate for 5 min before euthanizing the mice. Open the neck to expose the trachea and slowly inject 4 ml of PBS solution into the airways using a 20 ml syringe with a 7‐gauge needle, repeating this process 5 times. Collect the bronchoalveolar lavage fluid (BALF) using a flow cytometry tube and centrifuge at 300 g for 5 min to remove the supernatant. Incubate the cells with the diluted CD45 antibody on ice for 45 min. Use a flow cytometer to sort out cells that express the characteristic marker CD11b + . Cells were cultured in high glucose DMEM medium (#12491023, Gibco, ThermoFisher), supplemented with 1% PS (#15140148, Gibco, ThermoFisher) and 10% FBS (#10099141, Gibco, ThermoFisher), and maintained at 37°C in a 5% CO_2_ atmosphere.

### 2.2 Plasmid Transfection

The ALKBH5 over‐expression and knockdown plasmids were constructed and generated by Genelily Biotech Inc. (Shanghai, China). The shRNA‐scramble was used as negative control for transfection. Seed passaged macrophages onto a 6‐well plate at a density of 5×10^4 cells per cm² and culture them in DMEM + 10% FBS overnight until the cell confluency reaches 90%. After removing the culture medium, wash the cells twice with PBS, then add 1.5 ml of OPTI‐MEM (#A4124801, Gibco, ThermoFisher) transfection medium to each well and store in a 5% CO_2_, 37°C incubator. In two separate Eppendorf tubes containing 250ul of OPTI‐MEM, dilute 10ul of Lipofectamine 2000 (#11668030, Invitrogen, ThermoFisher) in one tube and 4ug of plasmid DNA expressing ALKBH5 overexpression or knockdown plasmids. After incubating at room temperature, mix the diluted solutions with the stored cell suspension and continue incubating in the 5% CO2, 37°C incubator for 5 h.

### 2.3 RNA Isolation and Quantitative‐Polymerase Chain Reaction (qPCR)

Total RNA was isolated using the RNeasy Plus Mini Kit (#74136, QIAGEN, Hilden, Germany) following the manufacturer's instructions. The concentration of total RNA was determined using a Nanodrop (NanoDrop1000, Thermo Fisher Scientific). For cDNA synthesis, 1 µg of total RNA was used with either the ReverTra Ace qPCR RT Master Mix (#FSQ‐201, TOYOBO) or SuperScript III Reverse Transcriptase (#1080044, Thermo Fisher Scientific), following the manufacturer's instructions. qPCR reactions were conducted on a Step One Plus Real‐Time PCR System (Applied Biosystems, Thermo Fisher Scientific) using the THUNDERBIRD qPCR Mix (#QPS‐201, TOYOBO, Osaka, Japan). The relative expression levels of RNA were determined using the ΔΔCt method, with normalization to glyceraldehyde 3‐phosphate dehydrogenase (GAPDH) mRNA. The specificity of all amplicons was confirmed through agarose gel visualization and/or melting curve analysis. Primer sequences are listed in Supplementary Table 1. Three biological replicates (independent cell isolations/mice) with technical duplicates were used.

### 2.4 Western Blotting

To extract total protein lysates from whole cells, 1× sodium dodecyl sulfate (SDS) sample buffer was used. The protein concentration was determined using the Pierce BCA Protein Assay Kit (#P0010, Beyotime, Shanghai, China). SDS‐polyacrylamide gel electrophoresis was performed to separate all proteins, which were then transferred to PVDF Blotting Membrane (IPVH00010, Merck Millipore, USA) using the Trans‐Blot Turbo Cassette (Bio‐Rad, Hercules, CA, USA). Blocking was carried out using Blocking One (#03953, Nacalai, Kyoto, Japan) or 5% skimmed milk. Primary antibodies for ALKBH5 (1:1000 dilution, #A11684; Abclonal), ULK1 (1:1000 dilution, #A8529; Abclonal), LC3 (1:1000 dilution, #A19665; Abclonal) and GAPDH (1:1000 dilution, #AC001; Abclonal) were incubated overnight at 4°C. Secondary antibodies for rabbit (1:10000 dilution, #AS014, Abclonal) were incubated with 1–5% skimmed milk at room temperature for 1 h. ChemiDocTouch (Bio‐Rad) was used to visualize the protein bands using Enhanced chemiluminescence (Pierce ECL Plus Substrate or West Atto Ultimate Sensitivity Substrate, Thermo Fisher Scientific). Three biological replicates (independent cell isolations/mice) with technical duplicates were used. Controls included: scrambled shRNA (negative), Torin1 (autophagy induction positive), Bafilomycin A1 (autophagy flux inhibition) were used to evaluate the atuphagy.

### 2.5 Dot Blot

RNA that had been preheated was applied to a positively charged nylon membrane (#FFN10, Beyotime, China) for the dot blot. The m6A status was probed using an m6A antibody and detected with ECL detection reagents (#WBULS0100, Millipore, USA). Additionally, the same membrane was stained with methylene blue as a control. In the case of immunofluorescence analysis, fixed cells were subjected to treatment with an m6A antibody followed by an HRP‐conjugated secondary antibody. The resulting outcomes were visualized using an imaging system (T5800, Tannon, Shanghai, China). Three biological replicates (independent cell isolations/mice) with technical duplicates were used.

### 2.6 M6A Immunoprecipitation

The Magna MeRIP™ m6A Kit (#17‐10499, Millipore, USA) was utilized to conduct the MeRIP assay, which aimed to determine the m6A modification of individual transcripts. In brief, total RNA (5 µg) was extracted from pretreated cells and then randomly fragmented to approximately 100 nucleotides in size. Immunoprecipitation of the RNA samples was performed using magnetic beads precoated with either anti‐m6A antibody or anti‐IgG as a control. To elute the m6A‐modified RNA fragments for further RT‐qPCR analysis, N6‐methyladenosine 5'‐monophosphate sodium salt was applied. Specific primers, designed according to the SRAMP website (m6A loci predictor: http://www.cuilab.cn/sramp/), were used for RT‐qPCR analysis. The relative enrichment of m6A was normalized to the input. The primer sequence were listed in the Supplementary Table 1. Three biological replicates (independent cell isolations/mice) with technical duplicates were used.

### 2.7 Immunofluorescence Analysis

For immunofluorescence analysis, cells were cultured on coverslips and fixed with 4% paraformaldehyde. Subsequently, permeabilization was carried out using 0.1% Triton X‐100. After blocking with 5% bovine serum albumin in PBS for 1 h at room temperature, the cells were incubated with primary antibodies against ALKBH5 (#HPA007196, Atlas Antibodies). Following this, a Goat anti‐Rabbit IgG (H + L) Cross‐Adsorbed Secondary Antibody, Alexa Fluor 546 (#A‐11010, Thermo Fisher Scientific, Waltham, MA, USA) was applied and allowed to incubate. To visualize the nuclei, ProLong® Gold Antifade Reagent with DAPI (#8961, CST) was used. The cells were then imaged using fluorescence microscopy with *z*‐stack image reconstructions conducted on the BZ‐9000 system (Keyence, Osaka, Japan). Three biological replicates (independent cell isolations/mice) with technical duplicates were used.

### 2.8 Transwell Migration Assay

To evaluate cell migration, a 24‐well plate with cell culture inserts (#353097, Falcon, Mexico City, Mexico) containing a filter with 8 μm‐diameter pores was used. Initially, the cells were serum‐starved for 24 h in RPMI1640 medium containing 0.1% FBS. Then, 1 × 10^5^ cells resuspended in 500 μL of RPMI1640 medium (#11879020, Gibco, Thermo Fisher) were seeded into the upper chamber of the insert. In the lower compartment of the chamber, RPMI1640 medium containing 10% FBS was added. Following an incubation period of 16 h, non‐migrating cells on the upper surface of the membrane were removed by wiping with a cotton‐tipped applicator. The migrating cells on the lower surface were fixed using cold methanol and stained with 0.5% crystal violet. The Hybrid Cell Count software (BZ‐II Analyzer, Keyence, Osaka, Japan) was utilized to automatically count the migrating cells in three random microscopic fields. Three biological replicates (independent cell isolations/mice) with technical duplicates were used.

### 2.9 Wound‐Healing Assays

To evaluate cell migration, cells were seeded at a density of 2 × 10^5^ cells in 6‐well plates. The cells were then incubated at 37°C with 5% CO_2_ for 48 h, followed by an additional 24‐h incubation in RPMI1640 medium containing 0.1% FBS. Subsequently, a wound was created by scratching the cell monolayer using a 200‐μL plastic tip. The cells were gently washed with PBS, and then incubated in RPMI1640 medium containing 10% FBS. The relative distance between the edges of the wound was observed at 3–6 time points after scratching, using an optical microscope (CKX53, Olympus, Tokyo, Japan). The assessment of the wound healing was performed using Image J software. Three biological replicates (independent cell isolations/mice) with technical duplicates were used.

### 2.10 Construction of ARDS Mouse Model

The experimental animals used in this study were SPF C57BL/6 strain male mice, aged 6–8 weeks and weighing 18–22 g. The mice were obtained from a supplier and allowed to acclimate for at least 3 days with free access to food and water. The mice were divided into six groups: a normal group (NC) without any treatment, a model treated with normal saline, and groups treated with lipopolysaccharide (LPS). Intratracheal LPS administration was selected to directly induce pulmonary inflammation, mimicking the local lung injury characteristic of ARDS, as opposed to systemic inflammation via intraperitoneal injection. LPS was purchased from the Selleck company (#S7850, Shanghai, China). Physiological saline was prepared at 60°C and kept at 37°C for later use. The NC group did not undergo any procedures. Mice were subjected to invasive tracheotomy intubation using a dose of 5 mg/kg per mouse. The mice were anesthetized using a small animal anesthesia machine and positioned supine on a wooden board with a 50° tilt angle. The submaxillary skin was incised, and the trachea was exposed. Using a syringe, 100 μl of air was pre‐inhaled, followed by slow injection of 100 μl of LPS solution or normal saline into the trachea. After the injection, the mice were gently rotated vertically to ensure even distribution of the drug in the lungs. The incision was sutured, and the mice were placed on a thermal insulation plate to recover from anesthesia. The mice were housed individually according to the requirements of each group. All procedures were approved by the Animal Care and Control Committee of Shanghai Chengxi Biotechnology Co. Ltd. (Protocol No: CX050701069) and adhered to guidelines of NIH and The Third Affiliated Hospital of Soochow University. Five biological replicates (independent cell isolations/mice) with technical duplicates were used.

### 2.11 Hematoxylin and Eosin (HE) Staining

The method of HE staining for lung sections in this study was based on a previous report. The following steps were followed for dehydration using an automatic dehydrator: 75% alcohol for 4 h, 85% alcohol for 2 h, 95% alcohol for 1 h, 100% alcohol for 0.5 h (repeated 5 times), xylene for 10 min (repeated twice), and paraffin for 1 h, 2 h, and 3 h. After dehydration, the lung sections were stained with hematoxylin and eosin (HE) for 10–20 min. Subsequently, the sections were rinsed with running water for 1–3 min, differentiated with alcohol hydrochloride for 5–10 s, and rinsed again with running water for 1–3 min. To return the blue color, the sections were placed in warm water at 50°C or a weak alkaline aqueous solution. After rinsing with running water for 1–3 min, the sections were treated with 85% alcohol for 3–5 min, stained with eosin for 3–5 min, and rinsed briefly for 3–5 s. Following staining, the sections were dehydrated using gradient alcohol, underwent xylene for transparent treatment, and sealed with neutral gum. Pannoramic 250 digital section scanner was used to collect images of the sections. The sections were initially observed at 40× magnification to identify macroscopic lesions. In selected areas, images were captured at 100× and 400× magnification to examine specific lesions. Lung injury histology score by two pathologists using a published system (0–4 for edema, inflammation, hemorrhage, necrosis).

### 2.12 ELISA

Enzyme‐Linked Immunosorbent Assay (ELISA) was performed to quantify the levels of inflammatory factors (IL‐1β, IL‐6, IL‐17A, and TNF‐α) in the culture medium supernatants of HASMCs. ELISA kits from R&D (#MLB00C, #M6000B, #M1700, #MTA00B, MN, USA) were utilized for this purpose. The optical density (OD) at a wavelength of 450 nm was measured using the Infinite F50® microplate reader manufactured by Tecan (CH). Three biological replicates (independent cell isolations/mice) with technical duplicates were used.

### 2.13 GFP‐LC3 Transfection and Autophagosome Quantification

For visualization and quantification of autophagosomes, alveolar macrophages were transfected with GFP‐LC3 plasmid (#24920, Addgene) using Lipofectamine 2000 according to the manufacturer's instructions. Briefly, cells were seeded on glass coverslips in 24‐well plates at a density of 1×10⁵ cells per well. After 24 h, cells were transfected with 1 µg GFP‐LC3 plasmid using 2 µl Lipofectamine 2000 in OPTI‐MEM medium. Following 6 h of transfection, the medium was replaced with complete DMEM containing 10% FBS. After 48 h of transfection, cells were treated with LPS (100 ng/mL) for 6 h. Where indicated, Torin1 (100 nM, #S2827, Selleckchem) was used as a positive control for autophagy induction, and Bafilomycin A1 (100 nM, #S1413, Selleckchem) was added for the final 4 h to inhibit autophagosome‐lysosome fusion. Cells were then fixed with 4% paraformaldehyde for 15 min at room temperature, permeabilized with 0.1% Triton X‐100 for 10 min, and blocked with 5% BSA for 1 h. Nuclei were counterstained with DAPI (#8961, CST) for 10 min. Coverslips were mounted using ProLong Gold Antifade reagent (#P36930, Thermo Fisher). Images were captured using a laser scanning confocal microscope (LSM 880, Zeiss) with a 63× oil immersion objective. For each condition, at least 50 cells from three independent experiments were analyzed. GFP‐LC3 puncta were quantified automatically using ImageJ software (NIH) with the following parameters: puncta size 0.5–2.0 µm², intensity threshold twofold above cytoplasmic background. Results are expressed as mean puncta number per cell ± SD.

### 2.14 Statistical Analysis

Statistical analysis was performed using SPSS 21.0 software (IBM Corp.) and data are presented as the mean ± SD. Sample sizes (*n* = 5 mice/group for in vivo, *n* = 3 for in vitro) were based on prior power analysis (α = 0.05, β = 0.8) using pilot data. Data were tested for normality (Shapiro‐Wilk) and homogeneity of variance (Levene's test) before ANOVA. Statistical differences between groups were determined using one‐way ANOVAs, followed by a Tukey's post hoc test for multiple comparisons in data with > 2 groups. *p* < 0.05 was considered to indicate a statistically significant difference.

## 3 Results

### 3.1 ALKBH5 Is Critical to the LPS‐Reduced RNA m6A Methylation and Elevated ULK1/ATG13 Expression

**Figure 1:**
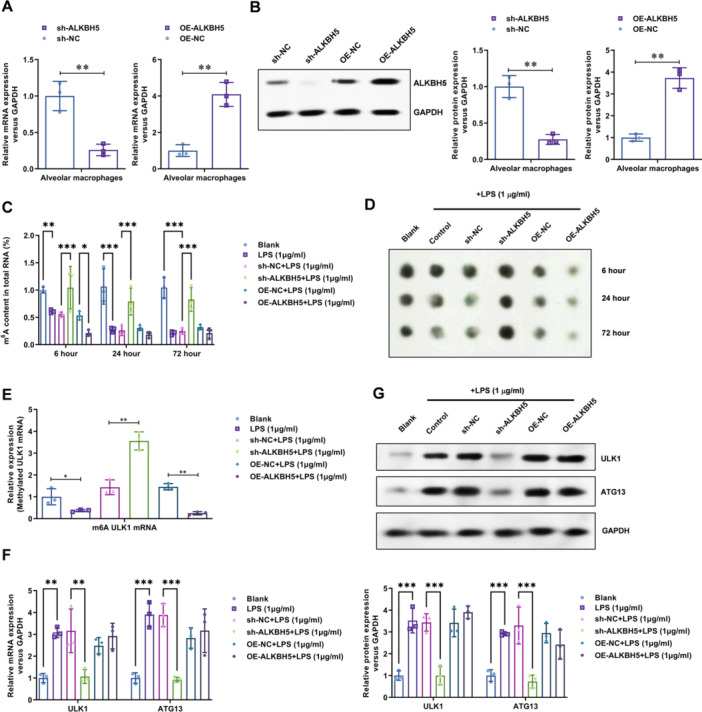
ALKBH5 is critical to the LPS‐reduced RNA m6A methylation and elevated ULK1/ATG13 expression. (A) Real‐time PCR was performed to assess the efficiency of ALKBH5 downregulation and overexpression in macrophages. (B) Western blot analysis was conducted to validate the efficiency of ALKBH5 downregulation and overexpression in macrophages. (C) A colorimetric assay was used to evaluate the impact of ALKBH5 downregulation and overexpression on total m6A levels in macrophages. (D) Dot blot assay was employed to verify the effect of ALKBH5 downregulation and overexpression on total m6A levels in macrophages. (E) Me‐RIP‐qPCR was employed to investigate the influence of ALKBH5 downregulation and overexpression on m6A methylation levels of ULK1 mRNA in macrophages. (F) Real‐time PCR was performed to examine the impact of ALKBH5 downregulation and overexpression on the mRNA levels of ULK1 and ATG13 in macrophages. (G) Western blot analysis was carried out to validate the effect of ALKBH5 downregulation and overexpression on the protein levels of ULK1 and ATG13 in macrophages. Data = mean ± SD; *n* = 3 group; *p < 0.05, **p < 0.01, ***p < 0.001 compared with indicated group, Statistical differences between groups were determined using one‐way ANOVAs, followed by a Tukey's post hoc test.

To investigate the regulatory role of ALKBH5 on alveolar macrophages in mice, we assessed the efficiency of ALKBH5 downregulation (*p* < 0.01) and overexpression (*p* < 0.01) using real‐time PCR (Figure 1A) and Western Blot (Figure 1B) to measure ALKBH5 mRNA and protein levels. The results indicated a significant decrease or increase in ALKBH5 expression, demonstrating its suitability for subsequent functional studies.

To study the regulation of RNA m6A levels in mouse alveolar macrophages by LPS stimulation and ALKBH5, we measured m6A levels in total RNA using m6A colorimetric assay (Figure 1C) and Dot Blot (Figure 1D). The results showed that ALKBH5 downregulation significantly increased m6A levels by 1.88 ± 0.21‐fold in total RNA (*p* < 0.001), while ALKBH5 overexpression significantly decreased m6A levels by 62 ± 5.68% (*p* < 0.01). We then performed MeRIP to enrich m6A‐modified RNA in mouse alveolar cells using lentiviral vectors for ALKBH5 downregulation or overexpression. Subsequently, we used real‐time PCR to detect changes in ULK1 mRNA levels. The results showed that LPS stimulation significantly inhibited m6A modification of ULK1 mRNA (*p* < 0.05), and ALKBH5 downregulation reversed this inhibition (*p* < 0.01) (Figure 1E). The expression levels of ULK1 mRNA and protein were detected using real‐time PCR (Figure 1E) and Western Blot (Figure 1G). The results demonstrated that LPS stimulation significantly upregulated the expression levels of ULK1 mRNA (*p* < 0.01) and protein (*p* < 0.001), while ALKBH5 downregulation reversed this upregulation. These findings suggest that ALKBH5 plays a critical regulatory role in alveolar macrophages by modulating m6A methylation levels and influencing the expression of target genes such as ULK1.

### 3.2 ALKBH5 Contributes to the LPS‐Induced Autophagy in Alveolar Macrophages

**Figure 2:**
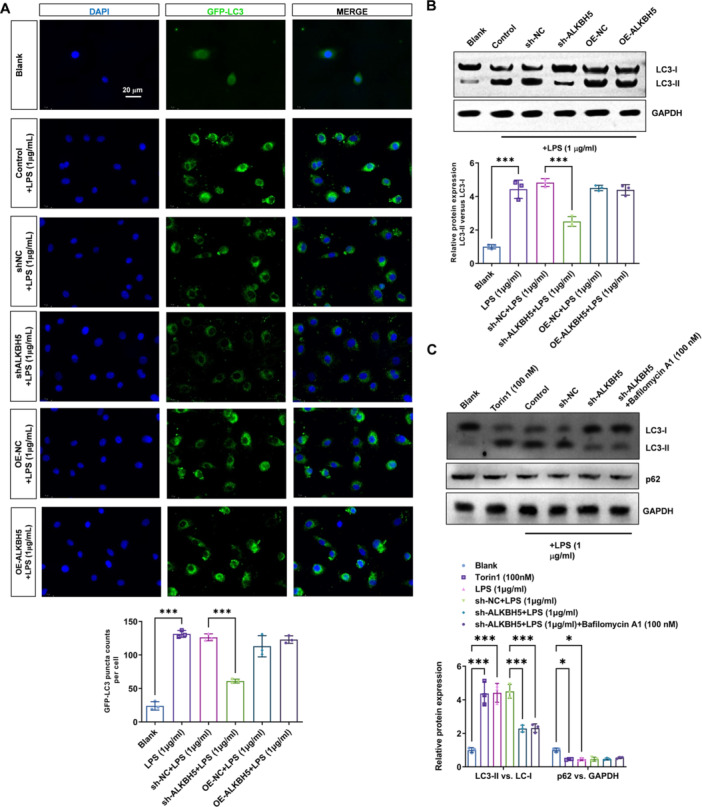
ALKBH5 contributes to the LPS‐induced autophagy in alveolar macrophages. (A) 200 × GFP‐LC3 images from laser confocal microscopy was used to investigate the impact of ALKBH5 downregulation and overexpression on autophagosome formation in macrophages, Scale bar=20 μm. (B) Western blot analysis was conducted to assess the effect of ALKBH5 downregulation and overexpression on the expression of autophagy protein LC3I/II in macrophages. (C) Western blot analysis was conducted to assess the effect of autophagy induction with Torin1 (100 nM), ALKBH5 downregulation and Bafilomycin A1 (100 nM) on the expression of autophagy protein LC3I/II and p62 in macrophages. Data = mean ± SD; n = 3 group; *p < 0.05, ***p < 0.001 compared with indicated group, Statistical differences between groups were determined using One‐way or Two‐way ANOVAs, followed by a Tukey's post hoc test.

LPS stimulation significantly upregulated autophagy levels in mouse alveolar cells, indicated by the GFP‐LC3 puncta per cell, while A ALKBH5 knockdown reduced puncta counts by ~62% (*p* < 0.001), supporting impaired autophagosome formation. (Figure 2A). Moreover, we measured the expression levels of the autophagy marker protein LC3‐I/II. We used GAPDH as the internal reference protein. The results demonstrated that LPS stimulation significantly upregulated the expression levels of the autophagy marker protein LC3‐I/II in mouse alveolar cells (*p* < 0.001), while ALKBH5 downregulation reversed this upregulation, decreased from 3.44 ± 0.40 to 1.05 ± 0.43 (*p* < 0.001) (Figure 2B). To further verified whether the ALKBH5 decreased LC3‐II/I result from either reduced autophagosome formation or increased autophagic degradation, we assess p62/SQSTM1 expression levels as an additional autophagy marker and use Bafilomycin A1 to differentiate between decreased LC3‐II due to reduced autophagosome formation versus increased degradation (Figure 2C). The positive control for autophagy was used with the Torin1 treatmentm, which showed a comparable increased LC3‐II/I (*p* < 0.001) and decreased p62 (*p* < 0.05). Moreover, Bafilomycin A1 treatment neither t affected the ALKBH5 decreased LC3‐II/I levels nor the p62 levels. These findings provide evidence that ALKBH5 regulates autophagy levels in mouse alveolar macrophages by the reduction of autophagosome formation. ALKBH5 knockdown suppresses LPS‐induced autophagy, mostly the autophagosome formation, confirming its role as a key autophagy regulator.

### 3.3 ALKBH5 Contributes to the LPS‐Induced M1 Polarization and Activated Migration and Invasion Ability in Alveolar Macrophages

**Figure 3:**
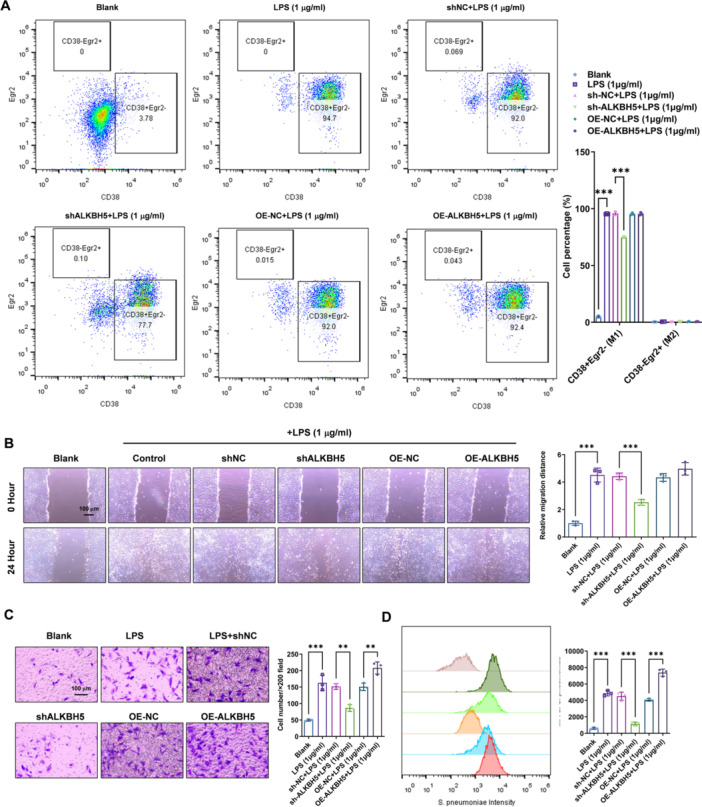
ALKBH5 contributes to the LPS‐induced M1 polarization and activated migration and invasion ability in alveolar macrophages. (A) ALKBH5 downregulation significantly reduced the number of M1 macrophages (CD38+Egr2‐) induced by LPS, while ALKBH5 overexpression had no significant effect. (B) 40× images from migration assay were performed to assess the impact of ALKBH5 downregulation and overexpression on macrophage migration levels, Scale bar=100 μm. (C) 40× images from Transwell chamber assay were conducted to evaluate the effect of ALKBH5 downregulation and overexpression on macrophage migration levels, Scale bar=100 μm. (D) Phagocytosis of FITC‐labeled S. pneumoniae (MOI 10:1; 2 h incubation). Flow cytometry analysis was used to measure the phagocytic capacity of mouse alveolar macrophages against Streptococcus pneumoniae. Data = mean ± SD; *n* = 3 group; **p < 0.01, ***p < 0.001 compared with indicated group, Statistical differences between groups were determined using one‐way ANOVAs, followed by a Tukey's post hoc test.

To investigate the effect of ALKBH5 on the differentiation and migration ability of mouse alveolar macrophages, we conducted the following experiments. Firstly, we used flow cytometry to determine the proportion of M1 (CD38+Egr2 ‐ ) and M2 (CD38+Egr2 + ) cells in mouse alveolar cells. The results showed that LPS stimulation significantly increased the proportion of M1 cells in mouse alveolar cells (*p* < 0.001), while downregulation of ALKBH5 reversed the increase in M1 cell proportion induced by LPS stimulation (*p* < 0.001) (Figure 3A). ALKBH5 knockdown reduced M1 but did not increase M2, as LPS polarizes macrophages toward M1 dominance. Secondly, we performed a scratch assay to evaluate the migration ability of mouse alveolar cells. The results demonstrated that LPS stimulation significantly enhanced the migration ability of mouse alveolar cells (*p* < 0.001), while downregulation of ALKBH5 reversed the increase in migration ability induced by LPS stimulation (*p* < 0.001) (Figure 3B). Additionally, we used Transwell chamber migration assay to assess the migration ability of mouse alveolar cells. The results showed that LPS stimulation significantly increased the migration ability of mouse alveolar cells (*p* < 0.01), while downregulation of ALKBH5 reversed the increase in migration ability induced by LPS stimulation (*p* < 0.01) (Figure 3C). Furthermore, we conducted a flow cytometry experiment to measure the phagocytic ability of mouse alveolar cells towards Streptococcus pneumoniae. The results showed that LPS stimulation significantly increased the phagocytic ability of mouse alveolar cells towards Streptococcus pneumoniae (*p* < 0.001), while downregulation of ALKBH5 reversed the LPS‐induced enhancement of phagocytic ability (*p* < 0.001) (Figure 3D). In summary, these findings suggest that ALKBH5 regulates the differentiation and migration ability of mouse alveolar macrophages.

### 3.4 ALKBH5 Contributes to the LPS‐Induced ARDS Inflammatory Response

**Figure 4:**
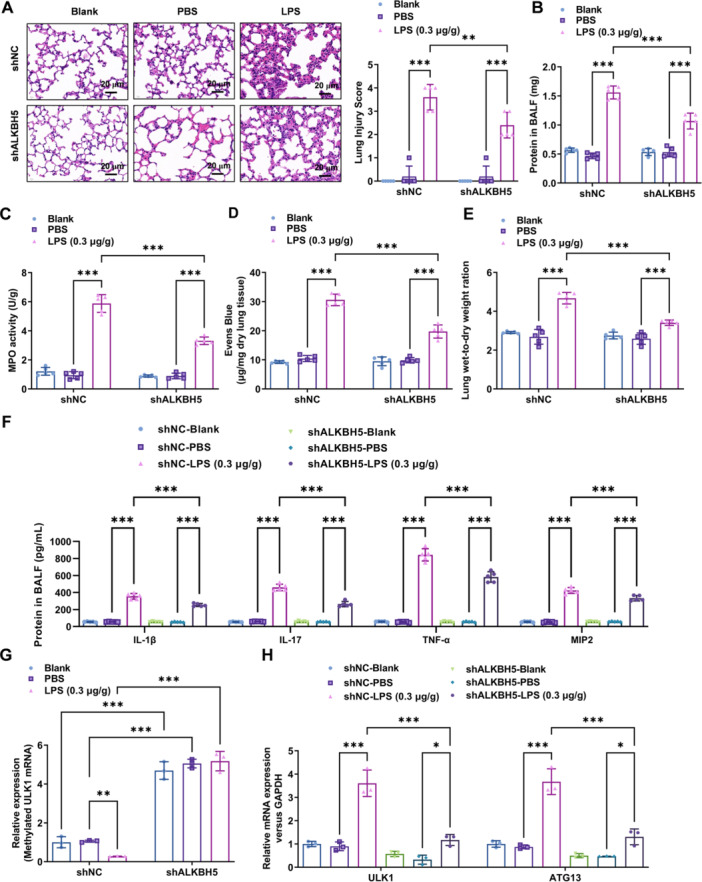
ALKBH5 contributes to the LPS‐induced ARDS inflammatory response. (A) In vivo mouse experiments were conducted to validate the role of ALKBH5 downregulation in alleviating ARDS‐associated inflammatory response. 100 × images from H&E staining was performed to assess lung tissue damage in mice, Scale bar=20 μm. (B–E) Biochemical indicators including protein levels in bronchoalveolar lavage fluid, MPO activity, Evans blue permeability, and lung edema were measured to confirm the effect of ALKBH5 downregulation in reducing ARDS‐related inflammation. (F) ELISA was utilized to detect the levels of inflammatory factors (IL‐1β, IL‐17, TNF‐α, and MIP2) in the bronchoalveolar lavage fluid of mice. (G–H) MeRIP and Real‐time PCR were employed to examine the (G) m6A levels and (H) total expression levels of ULK1 mRNA in mouse alveolar cells. Data = mean ± SD; *n* = 3 group; *p < 0.05, **p < 0.01, ***p < 0.001 compared with indicated group, Statistical differences between groups were determined using one‐way ANOVAs, followed by a Tukey's post hoc test.

To further validate the role of ALKBH5 downregulation in alleviating the inflammatory response in ARDS through in vivo mouse experiments, we found that downregulation of ALKBH5 significantly mitigated lung tissue damage in ARDS mice (*p* < 0.01) (Figure 4A). Further examination revealed that ALKBH5 downregulation markedly reduced protein levels in the bronchoalveolar lavage fluid of ARDS mice (*p* < 0.001) (Figure 4B), as well as myeloperoxidase (MPO) activity (*p* < 0.001) (Figure 4C), microvascular permeability (Figure 4D), and pulmonary edema (*p* < 0.001) (Figure 4E). ELISA experiments further demonstrated that ALKBH5 downregulation significantly decreased the levels of inflammatory factors (IL‐1β, IL‐17, TNF‐α, and MIP2) in the bronchoalveolar lavage fluid of ARDS mice (*p* < 0.001) (Figure 4F). MeRIP‐qPCR experiments indicated that ALKBH5 downregulation significantly increased the m6A levels of ULK1 mRNA in alveolar macrophages of ARDS mice (*p* < 0.001) (Figure 4G), as well as the overall expression levels of ULK1 and ATG13 mRNA (*p* < 0.001) (Figure 4H). These results suggested that ALKBH5 contributes to the LPS‐induced ARDS inflammatory response in vivo murine ARDS model. ALKBH5‐driven ULK1 demethylation amplifies both autophagy and IL‐1β/TNF‐α release, unifying its role in ARDS pathogenesis.

## 4 Discussion

The study established that ALKBH5 plays a crucial regulatory role in alveolar macrophages by modulating RNA methylation levels and influencing the expression of target genes. The downregulation of ALKBH5 resulted in reduced m6A methylation levels in total RNA, while its overexpression increased m6A levels. This suggests that ALKBH5 acts as an important modulator of RNA methylation, potentially affecting RNA stability, translation, and protein expression.

Furthermore, ALKBH5 was found to contribute to LPS‐induced autophagy in alveolar macrophages [41]. As reviewed by Vitaliti et al. [56], ALKBH5‐mediated autophagy aligns with broader mechanisms of macrophage reprogramming in inflammatory diseases. We found that the downregulation of ALKBH5 reversed the upregulation of autophagy levels induced by LPS stimulation. This observation was supported by the modulation of autophagosome formation and the expression levels of the autophagy marker protein LC3‐I/II. These findings suggest that ALKBH5 is involved in the regulation of autophagy, a cellular process crucial for maintaining cellular homeostasis and promoting cell survival under stress conditions. In addition to autophagy, ALKBH5 was shown to influence M1 polarization, migration ability, and phagocytic activity of alveolar macrophages. ALKBH5 downregulation reversed the LPS‐induced increase in M1 cell proportion, migration ability, and phagocytic activity. Our data align with Zhu et al. showing ALKBH5 promotes inflammation in sepsis [34] and its anti‐oncogene role in lung cancer [35], suggesting context‐dependent functions. This highlights the regulatory role of ALKBH5 in shaping the functional plasticity of alveolar macrophages, particularly in response to inflammatory stimuli. The METTL3/ALKBH5 axis may form a feedback loop in inflammation, as reported in sepsis; whether this occurs in ARDS macrophages is an exciting future direction.

Importantly, in an in vivo mouse model of ARDS, ALKBH5 downregulation alleviated the inflammatory response. Lung tissue damage, protein levels in bronchoalveolar lavage fluid, myeloperoxidase activity, microvascular permeability, and pulmonary edema were all significantly reduced in mice with downregulated ALKBH5. Furthermore, the downregulation of ALKBH5 resulted in decreased levels of inflammatory factors in bronchoalveolar lavage fluid. These findings suggest that ALKBH5 may play a critical role in promoting the pro‐inflammatory response in ARDS. The study also demonstrated that ALKBH5 downregulation increased m6A methylation levels of ULK1 mRNA and upregulated the expression levels of ULK1 and ATG13 mRNA. ULK1 is a key regulator of autophagy initiation, while ATG13 is involved in autophagosome formation. The upregulation of ULK1 and ATG13 mRNA suggests that ALKBH5 may influence autophagy‐related gene expression through RNA methylation modulation.

**Figure 5:**
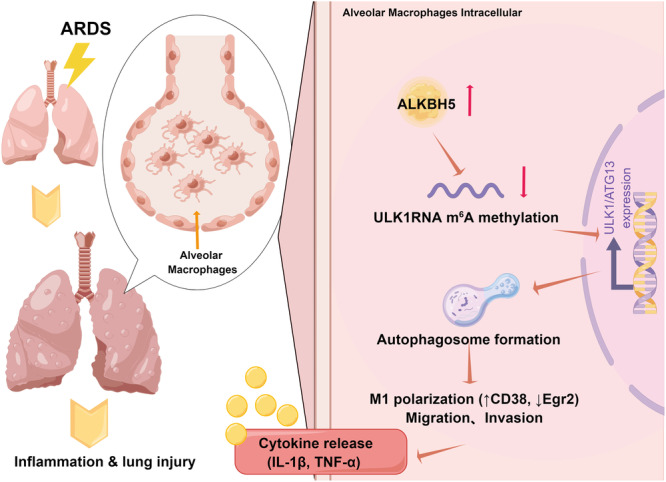
A schematic model showed that demonstrate that ALKBH5, an m6A RNA demethylase, plays a critical pathogenic role in ARDS. By erasing m6A modifications, ALKBH5 enhances ULK1/ATG13 expression, which concurrently boosts autophagy and promotes M1 pro‐inflammatory polarization in alveolar macrophages. This cascade ultimately amplifies the inflammatory response and exacerbates lung injury. Inhibition of ALKBH5 presents a promising novel therapeutic strategy for mitigating ARDS progression.

Overall, we demonstrate that ALKBH5‐mediated m6A demethylation of ULK1 contributes to macrophage autophagy and inflammation in ARDS, answering our central hypothesis (Figure 5). While our data suggest ALKBH5‐mediated demethylation stabilizes ULK1 mRNA, the precise mechanism (e.g., via YTHDF2 degradation complex) remains hypothetical and merits further investigation. Although ALKBH5 is elevated in human sepsis BALF, its ARDS‐specific expression requires validation. Inhibition risks (e.g., fertility, tumor suppression) may be mitigated by inhaled, macrophage‐targeted delivery. Pharmacological ALKBH5 inhibition merits clinical exploration as a disease‐modifying therapy. Targeting ALKBH5 may disrupt pathogenic m6A demethylation in ARDS, offering a novel therapeutic strategy. A deeper understanding of the molecular mechanisms underlying these processes could pave the way for the development of therapeutic strategies targeting ALKBH5 in the treatment of inflammatory lung diseases, such as ARDS. However, further researches, including (1) Murine model may not fully recapitulate human ARDS; (2) In vivo ALKBH5 inhibition was short‐term; (3) Unexamined crosstalk with METTL3. Future work will: (a) Validate findings in human alveolar macrophages; (b) Test ALKBH5 inhibitors (e.g., FB23‐2); (c) While MeRIP‐qPCR confirmed ULK1 as an m6A target, future studies employing MeRIP‐seq or site‐directed mutagenesis of predicted m6A motifs in ULK1 mRNA would precisely map functional sites; (d) Map ULK1 m6A sites via CRISPR‐RIP, are needed to clarify the precise mechanisms through which ALKBH5 modulates RNA methylation and the subsequent effects on alveolar macrophage function. (e) The association between ALKBH5‐mediated m6A erasure and ULK1 upregulation is supported by inverse methylation‐expression correlation, though direct causality requires future validation (e.g., ULK1 3'UTR m6A site mutagenesis). Additionally, exploring the potential interactions between ALKBH5 and other regulatory pathways involved in inflammation and immune responses would enhance our understanding of the intricate interplay within alveolar macrophage biology.

## Author Contributions

Gui Wang and Ting Zhou contributed equally to this work. Gui Wang and Ting Zhou performed the experiments, analyzed the data, and wrote the manuscript. Shujun Zhou designed, conceived and supervised the study, provided critical revisions to the manuscript, and acquired funding. All authors reviewed and approved the final manuscript.

## Ethics Statement

All animals were maintained in a pathogen‐free environment and used according to the approved protocols of the Animal Care and Control Committee of Shanghai Chengxi Biotechnology Co. Ltd. (CX050701069) and followed with the guidelines of NIH and The Third Affiliated Hospital of Soochow University.

## Conflicts of Interest

The authors declare no conflicts of interest.

## Supporting information

Supplementary Table 1: The sequences of the primers.

## Data Availability

All data obtained in this study are available from the corresponding author upon request.
