# Supplementary material for: ALKBH5‐Driven m6A Demethylation Boosts Inflammation and Autophagy in LPS‐Stimulated Macrophages
Source: Immun Inflamm Dis. 2026 Mar 16;14(3):e70348. doi: 10.1002/iid3.70348 (PMC13097382; doi:10.1002/iid3.70348)
Supplement: Supplementary file 1 — Supplementary Table 1: The sequences of the primers. [file IID3-14-e70348-s001.docx]

**Supplementary Table 1.** The sequences of the primers.

| **Gene** | **Forward primer (5’-3’)** | **Reverse primer (5’-3’)** |
| --- | --- | --- |
| Alkbh5 | GCGCGGTCATCAACGACTA | ATCAGCAGCATACCCACTGAG |
| Ulk1 | AAGTTCGAGTTCTCTCGCAAG | ACCTCCAGGTCGTGCTTCT |
| Atg13 | CCAGGCTCGACTTGGAGAAAA | AGATTTCCACACACATAGATCGC |
| GAPDH | AGGTCGGTGTGAACGGATTTG | GGGGTCGTTGATGGCAACA |
